# Geographic Distribution of Vaccinia Virus, Diagnosis and Demographic Aspects of Affected Populations, Minas Gerais, Brazil, 2000–2023

**DOI:** 10.3390/v17010022

**Published:** 2024-12-27

**Authors:** Pedro H. B. e Silva, Maycon D. de Oliveira, Iara M. de Almeida, Iago J. S. Domingos, Ana G. Stoffella-Dutra, Galileu Barbosa Costa, Jaqueline S. de Oliveira, Felipe C. M. Iani, Márcio R. de Castro, Jonatas S. Abrahão, Erna G. Kroon, Giliane de S. Trindade

**Affiliations:** 1 Instituto de Ciências Biológicas, Universidade Federal de Minas Gerais, Avenida Antônio Carlos, 6627, Belo Horizonte 31270-901, Brazil; flayner5@gmail.com (M.D.d.O.); iaraalmeida2301@gmail.com (I.M.d.A.); iagojsd@gmail.com (I.J.S.D.); anagstoffella@gmail.com (A.G.S.-D.); galileuk1@gmail.com (G.B.C.); jaquelinebmedica@hotmail.com (J.S.d.O.); jonatas.abrahao@gmail.com (J.S.A.); ernagkroon@gmail.com (E.G.K.); 2 Secretaria do Estado de Saúde de Minas Gerais, Cidade Administrativa, Rodovia Papa João Paulo II, Belo Horizonte 31585-200, Brazil; 3 Fundação Ezequiel Dias, Rua Conde Pereira Carneiro, 80, Belo Horizonte 30510-010, Brazil; felipe.iani@funed.mg.gov.br; 4 Clínica São Vicente, Avenida Japão, 309, Ipatinga 35160-118, Brazil; marcioinfecto@terra.com.br

**Keywords:** poxvirus, vaccinia virus, zoonosis, surveillance, occupational disease, epidemiology, outbreaks, public health

## Abstract

Since its first report in Brazil in 1999, outbreaks of exanthematous diseases caused by vaccinia virus (VACV) have been a recurring concern, particularly impacting rural regions. Minas Gerais (MG) State, Brazil, has emerged as the epicenter of bovine vaccinia (BV) outbreaks. This study presents a comprehensive overview of VACV circulation in MG State over the past two decades, examining the occurrence and distribution of poxvirus cases and outbreaks and the demographic characteristics of affected populations. Analysis of secondary databases from 2000 to 2023 revealed VACV circulation in at least 149 municipalities, particularly expanding in dairy regions. The study underscores BV as an occupational disease, predominantly affecting rural men involved in dairy cattle activities. Laboratory findings indicate high levels of anti-OPXV antibodies in most individuals, with some showing acute infections confirmed by qPCR testing. This analysis informs public health policies, emphasizing the need for enhanced surveillance of and preventive measures for dairy farming communities.

## 1. Introduction

The introduction of vaccinia virus (VACV) into Brazil is historically linked to its use in the global smallpox vaccination campaign, which ended in 1980, with Brazil concluding its efforts in 1978 [1,2]. The first documented outbreak of VACV in the country was reported in 1999, marking the emergence of bovine vaccinia (BV), a zoonotic disease that primarily affects dairy cattle and rural workers during milking and is classified as an occupational zoonosis [3,4,5,6].

The discontinuation of smallpox vaccination has led to an increased susceptibility of the global population to OPXV infections due to the loss of cross-protective immunity [1,7]. This vulnerability is exemplified by the recent multi-country mpox outbreak, highlighting the urgent need for improved epidemiologic surveillance, diagnostic tools and immunization strategies [8,9].

In Brazil, VACV circulates among a wide range of hosts, including rodents, marsupials and non-human primates, but cattle and humans remain the most important hosts [7,10]. Cattle act as major viral amplifiers, shed the virus through feces and milk, and facilitate its transmission to humans and other domestic animals. In particular, rural environments, especially dairy farms, appear to be critical sources of VACV transmission [11,12].

The state of Minas Gerais (MG), home to the largest dairy basin in Brazil, has been identified as the epicenter of BV outbreaks. Lesions in infected cows typically progress from red papules on the teats and udder to vesicles, pustules and finally ulcers before healing [13,14,15]. BV is not listed as a notifiable disease in Brazil, resulting in significant underreporting of VACV infections [16]. However, in states such as MG, where human cases of poxvirus are notifiable, data were available for this study [17]. Studies in endemic areas of MG have shown that healthcare professionals are often unaware of BV, although it is a notifiable disease [14,15,18,19]. This lack of knowledge may contribute to misdiagnosis and inadequate treatment, potentially leading to an underestimation of the true number of cases.

To comprehensively analyze the circulation of VACV in MG State and investigate the demographic aspects of the affected population, an observational, ecological time-series study was conducted using secondary data collected from different official databases. In addition, laboratory analyses of people affected by the disease in recent years were conducted.

## 2. Materials and Methods

### 2.1. Data Set

First, we used a database from Secretaria de Estado de Saúde de Minas Gerais (SES-MG), which comprises individual notifications of BV cases (bovines and humans) (Supplementary Table S1) and outbreak events (Supplementary Table S2) in the last two decades. Information on location, date of occurrence and demographic characteristics of the affected population were included. All data were recorded in accordance with the official standards of the Sistema de Informação de Agravos de Notificação (SINAN). We also used a second database from the Instituto Mineiro de Agropecuária (IMA), an animal defense agency of the state of MG, which contained notifications of bovine cases (Supplementary Table S3) in Minas Gerais from 2005 to 2017, offering detailed information on each occurrence location and date. To enrich our understanding of OPXV circulation in MG State, we conducted a bibliographic review (Supplementary Table S4) [4,13,14,15,20,21,22,23,24,25,26,27,28,29,30,31,32,33,34,35,36,37], encompassing articles describing serological detection, molecular detection or isolation of OPXV within the state. We considered only records with specific information, such as the municipality, date of sample collection and diagnosis for our analysis. Subsequently, we categorized evidence of detection based on the diagnostic criteria utilized (clinical, serological, molecular and isolation methods). For the descriptive analyses, all reports of the individual cases of BV by health professionals were considered, regardless of the laboratory diagnosis.

### 2.2. Geospatial Analysis, Agricultural and Livestock Production

For geospatial analysis of VACV cases analyses and comparisons with dairy production data in the state of Minas Gerais, we considered as evidence of circulation any report of poxvirus in a municipality. Each individual report may represent one or more diagnosed, confirmed or reported cases, with or without molecular, serological or isolated evidence, for both humans and animals.

Demographic census and agricultural and livestock production data were retrieved from the Brazilian Institute of Geography and Statistics (IBGE). We analyzed the demographic census from IBGE for the years 2000, 2010 and 2022 and the dairy production, cattle raising data and farm worker data from the IBGE agricultural and livestock census for the years 1995–1996, 2006 and 2017 [38,39,40,41,42,43] (Supplementary Table S5).

We compared the average values of each metric across all years with available data, as well as the mean per capita values based on the population data from the nearest year, with the accumulated case report data in order to provide a fair comparison since these reports occur in a similar 20-year period. Data for raw milk sales, cheese production and number of milked cows were not available for the years 1995–1996 and thus we only considered the data from 2006 and 2017 for these variables. Additionally, missing data may be present for some municipalities in one or two of the evaluated years for some of the variables; in these cases, we used the available data to calculate the mean.

All visual representations were generated in the R [44] programming language and statistical environment, with the help of the packages ggpplot2 [45], dplyr [46], sf [47,48], patchwork [49] and ggsn [50]. We used the package geobr to retrieve geospatial data for MG State and its municipalities provided by the Brazilian Institute for Applied Economic Research (IPEA) for the year 2020 [51].

### 2.3. Clinical Samples

The Laboratório de Vírus of the Universidade Federal de Minas Gerais has historically served as an auxiliary in the diagnosis of poxviruses with the SES. Therefore, 30 clinical samples from the years 2017 to 2021 were tested by the laboratory and included in the study. The clinical samples were obtained from 27 human individuals, corresponding to the notifications of individual cases of BV presented in the SES database from 10 municipalities of MG State. Of the 27 individuals, 3 had both crust and serum samples. One sample was obtained from Iturama (2019) and two samples were obtained from Joanésia (2021). Twenty individuals had only serum samples. One sample was obtained from Paracatu (2017), four from Salto da Divisa (2018), one from Prados (2019), eight from Joanésia (2021), four from Papagaios (2021), one from Juiz de Fora (2021) and one from Uberaba (2021). Additionally, four samples were obtained exclusively from crusts. Three samples were obtained from Teófilo Otoni (2018) and one from Araçuaí (2019).

### 2.4. Serological Assays

The serological diagnosis was carried out using the Plaque Reduction Neutralization Test (PRNT) assay, performed according to established protocols [52,53]. In brief, serum samples were diluted 1:20 and mixed with an equal volume of a virus suspension containing 150 PFU (plaque-forming units) of the VACV Western Reserve strain (VACV-WR), resulting in a final dilution of 1:40.

The mixture was homogenized and incubated overnight at 37 °C in a 5% CO_2_ atmosphere. Each plate included controls for infected and uninfected cells, with the addition of fetal bovine serum (FBS) (Gibco, Waltham, Massachusetts, USA) to ensure the viability of the viral control. BSC-40 cell monolayers in six-well plates were inoculated with the sample solutions and incubated for 48 h at 37 °C in a 5% CO_2_ atmosphere.

Once typical VACV-WR cytopathic effects were observed, all monolayers were fixed and stained with crystal violet. Samples were tested in duplicate, and the number of PFU in each well was counted. Positive sera were defined as those with PFU counts lower than 50% of the viral control. For titration, positive serum samples were serially diluted in a twofold ratio and tested as previously described. The antibody titer per sample was calculated based on the highest dilution showing a positive result and expressed in neutralizing units per milliliter (NU/mL).

### 2.5. Molecular Assays

To increase the sensitivity and specificity of molecular detection, we performed three distinct qPCR assays targeting fragments of the C11R [53], A56R [54] and E9L [55] genes on all clinical samples.

DNA extraction was performed on scab samples using the PureLink™ Genomic DNA Mini Kit (Invitrogen™, Carlsbad, CA, USA) according to the manufacturer’s instructions, while serum samples were diluted 1:10 and tested without DNA extraction. All reactions were carried out in 48-well plates using a StepOne instrument (Applied Biosystems, Foster City, CA, USA). The C11R and A56R reactions used the SYBR^®^ Green I Master Mix (Thermo Fisher Scientific, Waltham, MA, USA) and were considered positive if the melting temperature was within 1 °C of a positive control (DNA extracted from VACV-WR). The E9L reaction used the TaqMan^®^ Universal PCR Master Mix.

## 3. Results

### 3.1. Geographical Distribution of BV Cases Across MG State

**Figure 1:**
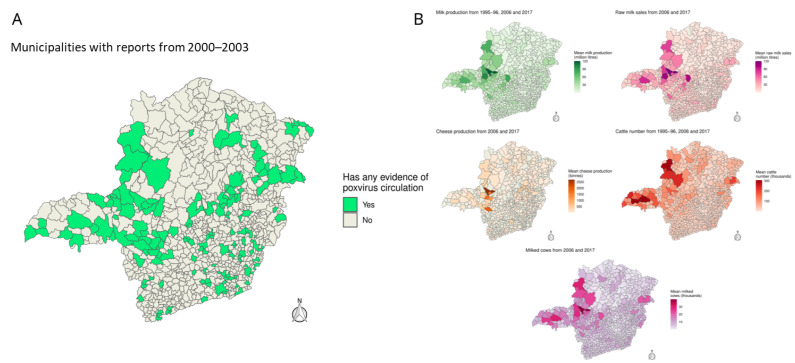
Poxvirus circulation in Minas Gerais associated with agricultural and livestock production. (**A**) In green municipalities that present at least one indicator of poxvirus circulation according to clinical or laboratory criteria from 2000 to 2023. (**B**) Municipal mean agricultural and livestock production from 1995 to 2017.

A total of 149 municipalities in MG State reported at least one case of BV, according to different detection and diagnosis criteria. There were at least 344 reports of BV cases in these municipalities affecting different host species, mostly in humans and dairy cattle. Analysis combined with IBGE data revealed a large overlap between areas with at least one indicator of VACV circulation and regions and municipalities with large numbers of cattle and dairy production (Figure 1).

In municipalities with reported BV cases, at least 53% exceed the 50th percentile of all analyzed variables. Notably, around 68% and 70% of these municipalities surpass the 50th percentile in raw milk sales and the number of milked cows, respectively, while 24% of the municipalities with documented cases are in the top 150 for all variables. Milk production, raw milk sales, cattle numbers and cheese production increased in most municipalities between the 2006 and 2017 agricultural censuses, though less than 45% saw growth in milked cows and cattle personnel. Among the top five milk-producing municipalities, there were four report cases: Patos de Minas, Patrocínio, Coromandel and Unaí. In contrast, only Belo Horizonte, among the bottom 30 milk producers, has case reports, which are from a 2016 outbreak (Supplementary Table S6). A similar trend is seen for the number of milked cows, with all top five municipalities reporting cases.

### 3.2. Descriptive Analysis of Infected Individuals

A total of 97 individual case notifications of bovine vaccinia in humans were recorded from 2001 to 2021 in MG State. It is important to note that the information is not uniformly distributed among all individuals due to the use of different databases with varying data availability. Most of the affected individuals were men (77/97, 79.38%), most were of working age (considering in this study 15–64 years old) (84/97, 86.6%) at the time of collection and most of the affected individuals should not have received the vaccine against smallpox (61/97, 62.89%), which was administered until 1978 in Brazil. Regarding ethnic composition, the majority self-reported themselves as mixed and white (30/63, 47.62%), and the habitation zones were predominantly rural (64/88, 72.63%) (Table 1). 

During the collection of epidemiological data, some notifications included different factors that could have increased the risk of exposure to VACV. Of all those who reported having had animal contact, all claimed to have had contact with cattle (35/35, 100%). Most individuals also reported contact with domestic animals (27/37, 72.97%), while a smaller proportion reported contact with wild animals (11/37, 29.73%) and rodents (4/37, 10.81%). The majority of respondents indicated that their exposure to VACV was directly related to their occupational activities (50/66, 70.76%), which involved direct contact with cattle.

Some individuals reported the occurrence of infection in other family members (8/37, 21.62%) (Table 2).

### 3.3. Laboratory Diagnosis

Among the 23 sera submitted to PRNT, 18 were positive for neutralizing anti-OPXV antibodies. Titers ranged from 50UN/mL to 800UN/mL (Table 3).

The 23 sera previously analyzed for PRNT with the addition of seven crust samples. No serum was positive in qPCR and all seven crusts were positive for all targets (Table 4).

Two seropositive individuals for PRNT from the municipality of Joanésia (ID 84,85) reside in the same household and engage in direct cattle work. A family nucleus of four seropositive individuals from the municipality of Papagaios, comprising a father, mother and two minor daughters (ID 93–96), in which the father is directly involved in cattle work. Two individuals positive for qPCR from the municipality of Teófilo Otoni (ID 67, 68) are siblings who work on different farms and became infected at the same time. Two individuals (ID 74, 82) from the municipalities of Iturama and Joanésia had serum and crust collected and were seronegative for PRNT. However, VACV DNA was detected in the crust samples, indicating primary exposure to VACV in unvaccinated individuals (based on age criteria).

## 4. Discussion

VACV is regarded as a neglected pathogen in Brazil, where it is not included in the list of mandatory notifiable diseases at the national level. As a result, BV cases are underreported, making it difficult to analyze the epidemiology of the virus in the country. MG is one of a few Brazilian states in which the notification of human cases of BV is mandatory. Consequently, the characterization of the state as the epicenter of the circulation of the disease may be biased, as the persistent circulation of VACV may be present in several regions of Brazil in which there are no notification records. Despite the notable increase in cases and outbreaks caused by VACV throughout the country, studies detecting the circulation of OPXV in Brazil have been predominantly conducted in states in the southeastern region of the country, such as MG State [4,6,7,10]. This suggests that another potential bias may exist in the areas where the virus is detected. The data compiled in this work demonstrate the evolution of the number of cases and municipalities affected by BV, with an overlap between VACV circulation areas and the largest dairy basins in MG State. The general population’s knowledge about OPXV and related diseases is very limited, and many health professionals working in endemic areas for VACV lack the necessary knowledge about BV to properly notify and manage patients [13,14,15,18]. As a result, underreporting by health agencies may be responsible for the lack of reported cases in several municipalities with large milk and dairy production. Therefore, it is quite possible that municipalities with large milk and dairy production, located near regions with evidence of detection, may also exhibit VACV circulation. The occupational nature of BV is reinforced by the results obtained in this study, with most affected individuals being working-age men who have direct contact with cattle and live in rural areas of the state. Additionally, this population sample shows that most individuals who work or live in areas at risk for VACV infection are susceptible to OPXV infections, as the vast majority were not old enough at the time of collection to have received the smallpox vaccine. The laboratory results presented show that most individuals have high titers of anti-OPXV neutralizing antibodies, which can indicate persistent exposure to VACV. Some individuals seronegative for PNRT had an acute infection at the time of collection, confirmed by molecular qPCR testing in the crust samples. These results indicate virus circulation in different human populations over the years, through direct or indirect evidence of circulation, and signal the increasing number of individuals susceptible to OPXV infections. This study also indicates possible familial VACV infections through laboratory analyses of households with different exposure profiles. It is important to note that most of the laboratory analyses were carried out on serum samples, which reduces the possibility of detecting active VACV infection in the samples, making it necessary to take samples of the lesion crust, when possible, for a more accurate diagnosis. This research provides valuable information on transmission dynamics and spread patterns within this region, which is essential for making informed decisions about implementing effective notification, control and prevention strategies. Considering the profile of affected individuals and the relationship with work, the possible inclusion of BV in the national list of work-related diseases could intensify epidemiological investigations from the point of view of occupational health surveillance.

## Figures and Tables

**Table 1:** Demographic and socioeconomic characteristics of the study population.

Characteristics	Group	(n)	%
Sex (n = 97)	Female	20	20.62
	Male	77	79.38
Age * (n = 97)	0–10 y/o	10	10.31
	11–20 y/o	12	12.37
	21–30 y/o	26	26.80
	31–40 y/o	22	22.68
	41–50 y/o	18	18.56
	>50 y/o	9	9.28
Working age ** (n = 97)	Yes	84	86.6
	No	13	13.4
Potentially vaccinated *** (n = 97)	Yes	31	37.11
	No	66	62.89
Ethnicity (n = 63)	White	30	47.62
	Mixed	30	47.62
	Black	3	4.76
Residential area (n = 88)	Rural	64	72.73
	Urban	24	27.27

* At the time of sample collection; ** 15–64 years old; *** Born before 1978.

**Table 2:** Risk factors to bovine vaccinia exposure.

Risk Factor	Yes (n)	%	No (n)	%
Contact with bovines (n = 38)	30	100	0	0
Contact with domestic animals * (n = 37)	27	72.98	10	27.02
Contact with wild animals ** (n = 37)	11	29.73	26	70.27
Contact with rodents (n = 37)	4	10.81	33	89.19
Work-related infection (n = 66)	50	70.76	16	24.24
Household infection (n = 37)	8	21.62	29	78.38

The sample size for each factor is indicated in parentheses. * Not including bovines; ** Not including rodents.

**Table 3:** Plaque reduction neutralization tests and titration of samples from clinical diagnosed individuals.

Report ID	County	Year *	Age **	Gender	Potentially Vaccinated	Titer (NU/mL)
48	Paracatu	2017	48	M	Yes	800
62	Salto da Divisa	2018	37	M	No	800
63	Salto da Divisa	2018	44	M	Yes	100
64	Salto da Divisa	2018	46	M	Yes	50
83	Joanésia	2021	48	M	Yes	400
84	Joanésia	2021	16	M	No	100
85	Joanésia	2021	20	M	No	50
86	Joanésia	2021	22	M	No	100
87	Joanésia	2021	43	M	No	50
88	Joanésia	2021	60	M	Yes	400
90	Joanésia	2021	24	M	No	200
91	Joanésia	2021	72	M	Yes	800
92	Juiz de Fora	2021	60	M	Yes	400
93	Papagaios	2021	30	M	No	800
94	Papagaios	2021	6	F	No	100
95	Papagaios	2021	28	F	No	100
96	Papagaios	2021	9	F	No	50
97	Uberaba	2021	40	M	No	400

* Year of sample collection; ** At the time of sample collection; (NU/mL) Neutralizing units per milliliter.

**Table 4:** Molecular detection of samples from clinically diagnosed individuals.

Report ID	County	Year *	Age **	Gender	Potentially Vaccinated
66	Teófilo Otoni	2018	30	M	No
67	Teófilo Otoni	2018	39	M	No
68	Teófilo Otoni	2018	41	M	Yes
69	Araçuaí	2019	30	M	No
74	Iturama	2019	42	M	Yes
82	Joanésia	2021	18	M	No
83	Joanésia	2021	48	M	Yes

* Year of sample collection; ** At the time of sample collection.

## Data Availability

Data are contained within the article and Supplementary Materials.

## Supplementary Materials

The following supporting information can be downloaded at: https://www.mdpi.com/article/10.3390/v17010022/s1, Table S1: Data base from Secretaria do Estado de Saúde of bovine vaccinia clinical diagnosed individuals; Table S2: Data base from Secretaria do Estado de Saúde of bovine vaccinia outbreaks reports; Table S3: Data base from Instituto Mineiro de Agropecuária of bovine vaccinia cases in bovines; Table S4: Bibliographical review of orthopoxvirus detection in Minas Gerais, Brazil; Table S5: Data on demographic census from IBGE for the years 2000, 2010 and 2022, dairy production, cattle raising, and farm worker from the IBGE agricultural and livestock census for the years 1995–1996, 2006 and 2017; Table S6: Data on milk-producing municipalities in Minas Gerais State.
