# Supplementary material for: Geographic Distribution of Vaccinia Virus, Diagnosis and Demographic Aspects of Affected Populations, Minas Gerais, Brazil, 2000–2023
Source: Viruses. 2024 Dec 27;17(1):22. doi: 10.3390/v17010022 (PMC11768858; doi:10.3390/v17010022)
Supplement: Supplementary file 1 [file viruses-17-00022-s001.zip › Supplementary Tables S1-S4.pdf]

Geographic distribution of vaccinia virus, diagnosis and demographic aspects of affected populations, Minas Gerais, Brazil, 2000-2023.

SUPPLEMENTARY TABLES

Supplementary Table S1. Data base from Secretaria do Estado de Saúde of bovine vaccinia clinical diagnosed individuals.

Report ID	County	Year	Age	Gender	Ethnicity	Residencial area	Potentially vaccinated	Work-related infection	Household infection	Animal contact			
										Bovine	Domestic	Rodent	Wild
1	Astolfo Dutra	2001	43	M	N/A	Urban	Yes	Yes	No	Yes	Yes	Yes	Yes
2	Piau	2001	44	M	N/A	Rural	Yes	Yes	No	Yes	Yes	Yes	Yes
3	Piau	2001	22	M	N/A	Rural	No	Yes	No	Yes	Yes	Yes	Yes
4	Tabuleiro	2001	16	M	N/A	Rural	No	Yes	No	Yes	Yes	Yes	Yes
5	Argirita	2008	45	M	N/A	Rural	Yes	Yes	No	Yes	No	No	No
6	Argirita	2008	22	M	N/A	Rural	No	Yes	No	Yes	No	No	No
7	Argirita	2008	37	M	N/A	Rural	Yes	Yes	No	Yes	No	No	No
8	Argirita	2008	34	M	N/A	Rural	Yes	Yes	No	Yes	Yes	No	No
9	Argirita	2008	32	F	N/A	Rural	Yes	No	No	Yes	Yes	No	No
10	Argirita	2008	42	M	N/A	Rural	Yes	Yes	No	Yes	No	No	No
11	Serro	2008	31	M	N/A	Rural	Yes	Yes	No	Yes	Yes	Yes	No
12	Serro	2008	29	M	N/A	Rural	No	Yes	No	Yes	Yes	Yes	No
13	Jordânia	2010	56	M	Parda	Rural	Yes	Yes	N/A	N/A	N/A	N/A	N/A
14	Jordânia	2010	29	M	Parda	Rural	No	Yes	N/A	N/A	N/A	N/A	N/A
15	Alvorada de Minas	2011	54	M	N/A	Urban	Yes	Yes	N/A	N/A	N/A	N/A	N/A
16	Serro	2011	19	M	N/A	Urban	No	N/A	N/A	N/A	N/A	N/A	N/A
17	Serro	2011	17	M	Parda	Rural	No	N/A	N/A	N/A	N/A	N/A	N/A
18	Serro	2011	53	M	Parda	Rural	Yes	N/A	N/A	N/A	N/A	N/A	N/A
19	Serro	2011	41	M	N/A	Rural	Yes	Yes	N/A	N/A	N/A	N/A	N/A
20	Serro	2011	31	M	N/A	Urban	No	Yes	N/A	N/A	N/A	N/A	N/A
21	Serro	2011	31	M	N/A	Rural	No	Yes	N/A	N/A	N/A	N/A	N/A
22	Serro	2011	25	M	White	Rural	No	Yes	N/A	N/A	N/A	N/A	N/A
23	Almenara	2011	36	M	N/A	Rural	Yes	No	N/A	N/A	N/A	N/A	N/A
24	Bandeira	2011	13	F	Parda	N/A	No	N/A	N/A	N/A	N/A	N/A	N/A

25	Bonfim	2011	40	F	White	N/A	Yes	N/A	N/A	N/A	N/A	N/A	N/A
26	Bonfim	2011	56	M	Parda	Rural	Yes	Yes	N/A	N/A	N/A	N/A	N/A
27	Bonfim	2011	27	M	White	N/A	No	N/A	N/A	N/A	N/A	N/A	N/A
28	Bonfim	2011	46	M	White	N/A	Yes	N/A	N/A	N/A	N/A	N/A	N/A
29	Bonfim	2011	25	M	White	Urban	No	N/A	N/A	N/A	N/A	N/A	N/A
30	Botelhos	2012	36	M	White	Rural	Yes	No	N/A	N/A	N/A	N/A	N/A
31	Cabo Verde	2012	31	F	White	Urban	No	N/A	N/A	N/A	N/A	N/A	N/A
32	Veríssimo	2012	10	F	White	Urban	No	N/A	N/A	N/A	N/A	N/A	N/A
33	Ibertioga	2013	14	F	Parda	Urban	No	N/A	N/A	N/A	N/A	N/A	N/A
34	Pingo-D'água	2014	29	F	Black	Rural	No	N/A	N/A	N/A	N/A	N/A	N/A
35	Santa Rita de Ibitipoca	2014	40	M	Parda	Rural	Yes	N/A	N/A	N/A	N/A	N/A	N/A
36	Santa Rita de Ibitipoca	2014	26	M	White	Rural	No	N/A	N/A	N/A	N/A	N/A	N/A
37	Santa Rita de Ibitipoca	2014	18	M	Parda	Rural	No	N/A	N/A	N/A	N/A	N/A	N/A
38	Santa Rita de Ibitipoca	2014	44	M	N/A	Urban	Yes	Yes	N/A	N/A	N/A	N/A	N/A
39	Santa Rita de Ibitipoca	2014	16	M	White	Rural	No	N/A	N/A	N/A	N/A	N/A	N/A
40	Santa Rita de Ibitipoca	2014	41	F	N/A	Rural	Yes	N/A	N/A	N/A	N/A	N/A	N/A
41	Santa Rita de Ibitipoca	2014	30	F	Parda	Rural	No	N/A	N/A	N/A	N/A	N/A	N/A
42	Santa Rita de Ibitipoca	2014	40	M	White	Rural	Yes	N/A	N/A	N/A	N/A	N/A	N/A
43	Santa Rita de Ibitipoca	2014	40	M	Parda	Urban	Yes	N/A	N/A	N/A	N/A	N/A	N/A
44	Santa Rita de Ibitipoca	2014	29	M	Parda	Rural	No	N/A	N/A	N/A	N/A	N/A	N/A
45	Santa Rita de Ibitipoca	2014	32	M	White	Rural	No	N/A	N/A	N/A	N/A	N/A	N/A
46	Pedra do Anta	2015	34	M	White	Urban	No	No	N/A	N/A	N/A	N/A	N/A
47	Santa Rita de Minas	2015	61	F	Parda	Rural	Yes	Yes	N/A	N/A	N/A	N/A	N/A
48	Paracatu	2017	48	M	N/A	Rural	Yes	Yes	No	Yes	Yes	No	No
49	Santa Rita de Minas	2017	6	M	Parda	N/A	No	N/A	N/A	N/A	N/A	N/A	N/A

50	Santa Rita de Minas	2017	23	M	Parda	Urban	No	Yes	N/A	N/A	N/A	N/A	N/A
51	Santa Rita de Minas	2017	48	M	Parda	N/A	Yes	No	N/A	N/A	N/A	N/A	N/A
52	Santa Rita de Minas	2017	23	M	White	Urban	No	N/A	N/A	N/A	N/A	N/A	N/A
53	Santa Rita de Minas	2017	38	F	White	N/A	No	No	N/A	N/A	N/A	N/A	N/A
54	Santa Rita de Minas	2017	23	F	White	Urban	No	Yes	N/A	N/A	N/A	N/A	N/A
55	Santa Rita de Minas	2017	25	M	White	N/A	No	No	N/A	N/A	N/A	N/A	N/A
56	Santa Rita de Minas	2017	17	F	White	Urban	No	N/A	N/A	N/A	N/A	N/A	N/A
57	Santa Rita de Minas	2017	6	F	White	Rural	No	N/A	N/A	N/A	N/A	N/A	N/A
58	Santa Rita de Minas	2017	46	M	White	Urban	Yes	No	N/A	N/A	N/A	N/A	N/A
59	Santa Rita de Minas	2017	26	F	Parda	Rural	No	N/A	N/A	N/A	N/A	N/A	N/A
60	Francisco Sá	2018	1	F	White	Rural	No	Yes	N/A	N/A	N/A	N/A	N/A
61	Salto da Divisa	2018	36	M	Parda	Urban	No	Yes	No	Yes	Yes	Yes	No
62	Salto da Divisa	2018	37	M	Parda	Urban	No	Yes	No	Yes	Yes	Yes	No
63	Salto da Divisa	2018	44	M	Parda	Urban	Yes	Yes	No	Yes	Yes	Yes	No
64	Salto da Divisa	2018	46	M	Parda	Rural	Yes	Yes	No	Yes	Yes	Yes	No
65	Salto da Divisa	2018	37	M	Parda	Urban	No	Yes	N/A	N/A	N/A	N/A	N/A
66	Teófilo Otoni	2018	30	M	Black	Rural	No	Yes	No	Yes	No	No	No
67	Teófilo Otoni	2018	39	M	Parda	Rural	No	Yes	Yes	Yes	No	No	No
68	Teófilo Otoni	2018	41	M	Parda	Rural	Yes	Yes	Yes	Yes	No	No	No
69	Araçuaí	2019	30	M	Parda	Rural	No	Yes	No	Yes	Yes	No	No
70	Conceição da Barra de Minas	2019	3	F	Parda	Rural	No	No	N/A	N/A	N/A	N/A	N/A

71	Conceição da Barra de Minas	2019	18	M	Preta	Rural	No	No	N/A	N/A	N/A	N/A	N/A
72	Itamarandiba	2019	27	M	N/A	N/A	No	N/A	N/A	N/A	N/A	N/A	N/A
73	Itamarandiba	2019	21	M	N/A	Rural	No	N/A	N/A	N/A	N/A	N/A	N/A
74	Iturama	2019	42	M	Parda	Urban	Yes	N/A	N/A	N/A	N/A	N/A	N/A
75	Prados	2019	28	M	White	Urban	No	Yes	N/A	Yes	N/A	N/A	N/A
76	Prados	2019	28	M	White	Rural	No	N/A	N/A	N/A	N/A	N/A	N/A
77	Novo Cruzeiro	2019	1	M	White	Urban	No	No	N/A	N/A	N/A	N/A	N/A
78	Itaipé	2020	3	M	Parda	Urban	No	No	N/A	N/A	N/A	N/A	N/A
79	Itaipé	2020	5	M	Parda	Rural	No	No	N/A	N/A	N/A	N/A	N/A
80	União de Minas	2020	54	M	White	Rural	Yes	Yes	N/A	N/A	N/A	N/A	N/A
81	Açucena	2021	40	M	Parda	Rural	No	Yes	N/A	N/A	N/A	N/A	N/A
82	Joanésia	2021	18	M	N/A	Rural	No	Yes	No	Yes	Yes	No	No
83	Joanésia	2021	48	M	N/A	Rural	Yes	Yes	No	Yes	Yes	No	No
84	Joanésia	2021	16	M	N/A	Rural	No	Yes	Yes	Yes	Yes	No	No
85	Joanésia	2021	20	M	N/A	Rural	No	Yes	Yes	Yes	Yes	No	No
86	Joanésia	2021	22	M	N/A	Rural	No	Yes	No	Yes	Yes	No	No
87	Joanésia	2021	43	M	N/A	Rural	Yes	Yes	No	Yes	No	No	No
88	Joanésia	2021	60	M	N/A	Rural	Yes	Yes	No	Yes	Yes	No	No
89	Joanésia	2021	41	M	N/A	Rural	No	Yes	No	Yes	No	No	No
90	Joanésia	2021	24	M	N/A	Rural	No	Yes	No	Yes	Yes	No	No
91	Joanésia	2021	72	M	N/A	Rural	Yes	Yes	No	Yes	Yes	Yes	No
92	Juiz de Fora	2021	60	M	N/A	Rural	Yes	Yes	No	Yes	Yes	No	No
93	Papagaios	2021	30	M	White	Rural	No	Yes	Yes	Yes	Yes	No	No
94	Papagaios	2021	6	F	White	Rural	No	No	Yes	Yes	Yes	No	No
95	Papagaios	2021	28	F	White	Rural	No	No	Yes	Yes	Yes	No	No
96	Papagaios	2021	9	F	White	Rural	No	No	Yes	Yes	Yes	No	No
97	Uberaba	2021	40	M	White	Rural	No	Yes	No	Yes	No	No	No

Supplementary Table S2. Data base from Secretaria do Estado de Saúde of bovine vaccinia outbreaks reports.

Report ID	County	Year	Cases
98	Além Paraíba	2008	1
99	Argirita	2008	11
100	Recreio	2008	2
101	Argirita	2009	2
102	Argirita	2009	2
103	Argirita	2009	3
104	Santa Rita de Jacutinga	2009	5
105	Pedrinópolis	2010	5
106	Pedrinópolis	2010	8
107	Pedrinópolis	2010	4
108	Pedrinópolis	2010	3
109	Pedrinópolis	2010	8
110	Pedrinópolis	2010	5
111	Pedrinópolis	2010	5
112	Itapagipe	2010	2
113	Bandeira	2010	11
114	Açucena	2011	1
115	Conceição da Barra de Minas	2013	11
116	Belo Horizonte	2016	34
117	Salto da Divisa	2018	5

Supplementary Table S3. Data base from Instituto Mineiro de Agropecuária of bovine vaccinia cases in bovines.

Report ID	County	Year	Species
118	Bambuí	2005	Bovine
119	Bambuí	2005	Bovine
120	Bambuí	2005	Bovine
121	Iguatama	2005	Bovine
122	Jequeri	2005	Bovine
123	Juiz de Fora	2005	Bovine
124	Manhumirim	2005	Bovine
125	Mariana	2005	Bovine
126	Medeiros	2005	Bovine
127	Mercês	2005	Bovine
128	Nova Era	2005	Bovine
129	Nova Era	2005	Bovine
130	Patrocínio do Muriaé	2005	Bovine
131	Rio Pomba	2005	Bovine
132	Sabinópolis	2005	Bovine
133	Santo Antônio do Itambé	2005	Bovine
134	Santo Antônio do Itambé	2005	Bovine
135	Santo Antônio do Itambé	2005	Bovine
136	Serro	2005	Bovine
137	Serro	2005	Bovine
138	Serro	2005	Bovine
139	Acaiaca	2006	Bovine
140	Antônio Prado de Minas	2006	Bovine
141	Bambuí	2006	Bovine
142	Bambuí	2006	Bovine
143	Carlos Chagas	2006	Bovine
144	Conceição do Mato Dentro	2006	Bovine
145	Córrego Danta	2006	Bovine
146	Córrego Danta	2006	Bovine
147	Dom Silvério	2006	Bovine
148	Guaraciaba	2006	Bovine
149	Iguatama	2006	Bovine
150	Iguatama	2006	Bovine
151	Iguatama	2006	Bovine
152	Itambacuri	2006	Bovine
153	Itapagipe	2006	Bovine
154	Jequeri	2006	Bovine
155	Malacacheta	2006	Bovine
156	Mariana	2006	Bovine

157	Mariana	2006	Bovine
158	Mariana	2006	Bovine
159	Mariana	2006	Bovine
160	Mariana	2006	Bovine
161	Mariana	2006	Bovine
162	Mariana	2006	Bovine
163	Martins Soares	2006	Bovine
164	Medeiros	2006	Bovine
165	Medeiros	2006	Bovine
166	Medeiros	2006	Bovine
167	Mercês	2006	Bovine
168	Pratinha	2006	Bovine
169	Rio Vermelho	2006	Bovine
170	Santo Antônio do Itambé	2006	Bovine
171	São João Evangelista	2006	Bovine
172	Serro	2006	Bovine
173	Tiros	2006	Bovine
174	Formiga	2007	Bovine
175	Itamarandiba	2007	Bovine
176	Mariana	2007	Bovine
177	Nanuque	2007	Bovine
178	Paulistas	2007	Bovine
179	Pimenta	2007	Bovine
180	Piranguçu	2007	Bovine
181	Sabinópolis	2007	Bovine
182	Wenceslau Braz	2007	Bovine
183	Wenceslau Braz	2007	Bovine
184	Argirita	2008	Bovine
185	Argirita	2008	Bovine
186	Carlos Chagas	2008	Bovine
187	Coromandel	2008	Bovine
188	Estrela do Sul	2008	Bovine
189	Gonçalves	2008	Bovine
190	Luislândia	2008	Bovine
191	Monte Carmelo	2008	Bovine
192	Paraguaçu	2008	Bovine
193	Paraguaçu	2008	Bovine
194	Paraisópolis	2008	Bovine
195	Porteirinha	2008	Bovine
196	Pratinha	2008	Bovine
197	Sapucaí-Mirim	2008	Bovine
198	Sapucaí-Mirim	2008	Bovine
199	Serro	2008	Bovine
200	Andradas	2009	Bovine

201	Andradas	2009	Bovine
202	Areado	2009	Bovine
203	Areado	2009	Bovine
204	Areado	2009	Bovine
205	Areado	2009	Bovine
206	Carmo do Paranaíba	2009	Bovine
207	Conquista	2009	Bovine
208	Doresópolis	2009	Bovine
209	Doresópolis	2009	Bovine
210	Doresópolis	2009	Bovine
211	Doresópolis	2009	Bovine
212	Doresópolis	2009	Bovine
213	Grupiara	2009	Bovine
214	João Pinheiro	2009	Bovine
215	João Pinheiro	2009	Equine
216	Lagoa Formosa	2009	Bovine
217	Matias Barbosa	2009	Bovine
218	Paraisópolis	2009	Bovine
219	Passos	2009	Bovine
220	Patos de Minas	2009	Bovine
221	Piumhi	2009	Bovine
222	Sabinópolis	2009	Bovine
223	Serra dos Aimorés	2009	Bovine
224	Tapira	2009	Bovine
225	Tapira	2009	Bovine
226	Tiros	2009	Bovine
227	Abadia dos Dourados	2010	Bovine
228	Caranaíba	2010	Bovine
229	Carmo do Paranaíba	2010	Bovine
230	Desterro do Melo	2010	Bovine
231	Felício dos Santos	2010	Bovine
232	Frutal	2010	Bovine
233	Ibiá	2010	Bovine
234	Ibiá	2010	Bovine
235	Itamarandiba	2010	Bovine
236	Itapagipe	2010	Bovine
237	Lagoa Formosa	2010	Bovine
238	Luz	2010	Bovine
239	Luz	2010	Bovine
240	Luz	2010	Bovine
241	Luz	2010	Bovine
242	Materlândia	2010	Bovine
243	Medeiros	2010	Bovine
244	Monte Carmelo	2010	Bovine

245	Patos de Minas	2010	Bovine
246	Patos de Minas	2010	Bovine
247	Patos de Minas	2010	Bovine
248	Patrocínio	2010	Bovine
249	Pedrinópolis	2010	Bovine
250	Rio Doce	2010	Bovine
251	Rio Vermelho	2010	Bovine
252	Sacramento	2010	Bovine
253	Sacramento	2010	Bovine
254	São Geraldo do Baixio	2010	Bovine
255	São Geraldo do Baixio	2010	Bovine
256	São José do Jacuri	2010	Bovine
257	São Roque de Minas	2010	Bovine
258	Tapira	2010	Bovine
259	Tiros	2010	Bovine
260	Tombos	2010	Bovine
261	Uberaba	2010	Bovine
262	Vargem Bonita	2010	Bovine
263	Vargem Bonita	2010	Bovine
264	Vargem Bonita	2010	Bovine
265	Vargem Bonita	2010	Bovine
266	Vargem Bonita	2010	Bovine
267	Vargem Bonita	2010	Bovine
268	Veríssimo	2010	Bovine
269	Veríssimo	2010	Bovine
270	Veríssimo	2010	Bovine
271	Açucena	2011	Bovine
272	Areado	2011	Bovine
273	Aricanduva	2011	Bovine
274	Belo Vale	2011	Bovine
275	Brumadinho	2011	Bovine
276	Grão Mogol	2011	Bovine
277	Guapé	2011	Bovine
278	Guarda-mor	2011	Bovine
279	Manhuaçu	2011	Bovine
280	Maravilhas	2011	Bovine
281	Marliéria	2011	Bovine
282	Piedade do Rio Grande	2011	Bovine
283	Prata	2011	Bovine
284	Santa Margarida	2011	Bovine
285	Santana de Pirapama	2011	Bovine
286	São Roque de Minas	2011	Bovine
287	São Roque de Minas	2011	Bovine
288	Serro	2011	Bovine

289	Serro	2011	Bovine
290	Serro	2011	Bovine
291	Serro	2011	Bovine
292	Tapira	2011	Bovine
293	Uberaba	2011	Bovine
294	Vargem Bonita	2011	Bovine
295	Ibiá	2013	Bovine
296	Mariana	2013	Bovine
297	Santa Luzia	2013	Bovine
298	Bom Jesus do Galho	2014	Bovine
299	Carmésia	2014	Bovine
300	Itaúna	2014	Bovine
301	São Gonçalo do Pará	2014	Bovine
302	São Pedro dos Ferros	2014	Bovine
303	Veríssimo	2014	Bovine
304	Abadia dos Dourados	2015	Bovine
305	Bom Despacho	2015	Bovine
306	Douradoquara	2015	Bovine
307	Igaratinga	2015	Bovine
308	Ladainha	2015	Bovine
309	Ladainha	2015	Bovine
310	Mariana	2015	Bovine
311	São Miguel do Anta	2015	Bovine
312	Vermelho Novo	2015	Bovine
313	Araguari	2016	Bovine
314	Carneirinho	2017	Bovine
315	Carneirinho	2017	Bovine
316	Santa Rita de Jacutinga	2017	Bovine

Supplementary Table S4. Bibliographical review of orthopoxvirus detection in Minas Gerais, Brazil.

Report ID	County	Year	Criteria	Source
317	Muriaé	2000	Isolation	TRINDADE et al., 2007
318	Guarani	2001	Isolation	TRINDADE et al., 2006
319	Passatempo	2003	Isolation	LEITE et al., 2005
320	Bambuí	2005	Isolation	ABRAHÃO et al., 2009
321	Mariana	2005	Isolation	ABRAHÃO et al., 2009
322	Paraguaçu	2005	Isolation	ABRAHÃO et al., 2009
323	Resplendor	2005	Isolation	ASIS et al., 2012
324	Serro	2005	Isolation	TRINDADE et al., 2016
325	Itajubá	2007	Eletronic microscopy	SILVA et al., 2008
326	Mariana	2008	Isolation	ABRAHÃO et al., 2009
327	Doresópolis	2010	Isolation	ABRAHÃO et al., 2015
328	Carangola	2010	PRNT	BORGES et al., 2017
329	Curvelo	2010	PRNT	BORGES et al., 2017
330	Serro	2010	PRNT	BORGES et al., 2017
331	Serro	2011	Isolation	ASIS et al., 2012
332	Sabará	2011	ELISA and PCR	MIRANDA et al., 2017
333	Serro	2011	PCR	REHFELD et al., 2018
334	Serro	2012	PCR and PRNT	COSTA et al., 2015
335	Belo Horizonte	2012	PCR	COSTA et al., 2017
336	Belo Horizonte	2012	PCR	DUTRA et al., 2017
337	Rio Pomba	2012	ELISA and PCR	MIRANDA et al., 2017
338	Serro	2012	ELISA and PCR	MIRANDA et al., 2017
339	Belo Horizonte	2013	PCR and PRNT	COSTA et al., 2018
340	Carangola	2015	Isolation	LIMA et al., 2018
341	Serro	2018	PRNT	OLIVEIRA et al., 2023
342	Coronel Murta	2020	PRNT	ABREU et al., 2022
343	Unaí	2021	PRNT	ABREU et al., 2022
344	Serro	2021	PRNT	DOMINGOS elt al., 2023
